# Selective Suppression of Local Interneuron Circuits in Human Motor Cortex Contributes to Movement Preparation

**DOI:** 10.1523/JNEUROSCI.2869-17.2017

**Published:** 2018-01-31

**Authors:** Ricci Hannah, Sean E. Cavanagh, Sara Tremblay, Sara Simeoni, John C. Rothwell

**Affiliations:** Sobell Department of Motor Neuroscience and Movement Disorders, UCL Institute of Neurology, London, United Kingdom

**Keywords:** corticospinal, inhibition, motor cortex, motor preparation, transcranial magnetic stimulation

## Abstract

Changes in neural activity occur in the motor cortex before movement, but the nature and purpose of this preparatory activity is unclear. To investigate this in the human (male and female) brain noninvasively, we used transcranial magnetic stimulation (TMS) to probe the excitability of distinct sets of excitatory inputs to corticospinal neurons during the warning period of various reaction time tasks. Using two separate methods (H-reflex conditioning and directional effects of TMS), we show that a specific set of excitatory inputs to corticospinal neurons are suppressed during motor preparation, while another set of inputs remain unaffected. To probe the behavioral relevance of this suppression, we examined whether the strength of the selective preparatory inhibition in each trial was related to reaction time. Surprisingly, the greater the amount of selective preparatory inhibition, the faster the reaction time was. This suggests that the inhibition of inputs to corticospinal neurons is not involved in preventing the release of movement but may in fact facilitate rapid reactions. Thus, selective suppression of a specific set of motor cortical neurons may be a key aspect of successful movement preparation.

**SIGNIFICANCE STATEMENT** Movement preparation evokes substantial activity in the motor cortex despite no apparent movement. One explanation for the lack of movement is that motor cortical output in this period is gated by an inhibitory mechanism. This notion was supported by previous noninvasive TMS studies of human motor cortex indicating a reduction of corticospinal excitability. On the contrary, our data support the idea that there is a coordinated balance of activity upstream of the corticospinal output neurons. This includes a suppression of specific local circuits that supports, rather than inhibits, the rapid generation of prepared movements. Thus, the selective suppression of local circuits appears to be an essential part of successful movement preparation instead of an external control mechanism.

## Introduction

Neural activity in motor cortex occurs not only during the execution of movement, but also in the preparatory period before movement (Tanji and Evarts, 1976; Riehle and Requin, 1989; Kaufman et al., 2014). However, the nature of this preparatory activity is still unclear. A common assumption, dating back to classic studies (Tanji and Evarts, 1976), is that it represents a subthreshold version of the activity that accompanies movement. The preparatory activity is prevented from generating movement by a presumed “gating” mechanism.

Initial experiments with transcranial magnetic stimulation (TMS) appeared to be consistent with this idea. Rather than finding a subtle increase in excitability during the preparatory period, as expected by the subthreshold hypothesis, many studies reported a paradoxical reduction (Hasbroucq et al., 1997; Touge et al., 1998; Duque and Ivry, 2009), which was originally interpreted as an inhibitory signal that prevents premature expression of premovement activity (Touge et al., 1998; Duque and Ivry, 2009). Effectively, corticospinal neurons were envisaged as being inhibited so that they could not respond to a gradually increasing amount of preparatory excitation. However, other explanations were also put forward. Hasbroucq et al. (1997) thought inhibition might increase the signal-to-noise ratio in motor cortex by suppressing unwanted inputs that were irrelevant to the task. Others suggested (Bestmann and Duque, 2016; Duque et al., 2017) that inhibition may be important in action selection, for example, by preventing certain inputs from driving a muscle in an inappropriate way. However, neither of these explanations addresses the question of why preparatory activity in motor areas is not accompanied by a detectable change in motor output.

The dynamical systems approach provides an alternative way of viewing preparatory activity. It analyzes the activity of populations of neurons without any assumptions about the particular role of individual cells. Individual neural firing rates are subsumed into a dynamically evolving population output. The approach highlights the fact that the activity of many single neurons is tuned differently in the preparatory and movement epochs, meaning that the preparatory activity cannot be a subthreshold version of the movement command (Churchland et al., 2010; Kaufman et al., 2010; Elsayed et al., 2016). Instead, it is suggested that preparatory activity represents a separate initial neural state that will evolve into the movement (Churchland et al., 2010; Kaufman et al., 2014; Elsayed et al., 2016). In this scenario, there is a balance of excitatory (and inhibitory) input to corticospinal neurons during the premovement period that facilitates preparation, but ultimately cancels out so that no movement occurs (Kaufman et al., 2014). The activity then evolves to produce a movement upon receipt of an imperative command (Kaufman et al., 2016). It is important to note that this population-based description of neural activity can in principle accommodate the idea that subpopulations behave according to a “signal-to-noise” or “action selection” hypothesis.

The purpose of the present experiments was to test the inhibitory gating version of the “subthreshold hypothesis.” At its simplest, this hypothesis predicts that an external inhibitory input prevents the release of an evolving excitatory corticospinal command. If this is true, then we predict that the corticospinal response to any facilitatory input ought to be suppressed. In contrast, if there is a patterned suppression of inputs, as predicted by the “dynamical systems hypothesis”, or the more nuanced versions of a subthreshold hypothesis, we may be able to demonstrate that only a proportion of these inputs is suppressed. A second prediction is that if inhibition prevents the premature release of movement, then less preparatory inhibition might be expected to speed movement onset. Alternatively, if inhibition is an essential part of preparatory activity, then we might expect movements to take longer to evolve when preparatory inhibition fails to occur.

We used novel TMS methods to activate two separate subsets of excitatory inputs that drive corticospinal neurons (D'Ostilio et al., 2016; Hannah and Rothwell, 2017). We could then examine whether each of these was suppressed to the same extent during movement preparation. In addition, we could ask whether the degree of suppression correlated, in each individual, with the reaction time (RT) on that trial. Finally, we tested whether movements requiring more explicit inhibition, such as within a Go/No Go task, have similar effects on corticospinal inputs.

## Materials and Methods

### 

#### Subjects

A total of 59 right-handed, healthy, human volunteers (30 males; mean age, 24 ± 1 years; age range, 19–42 years), who reported no contraindications to TMS (Rossi et al., 2011), provided written informed consent before participating in the study, which was approved by the University College London Ethics Committee.

#### Reaction time tasks

**Figure 1.**
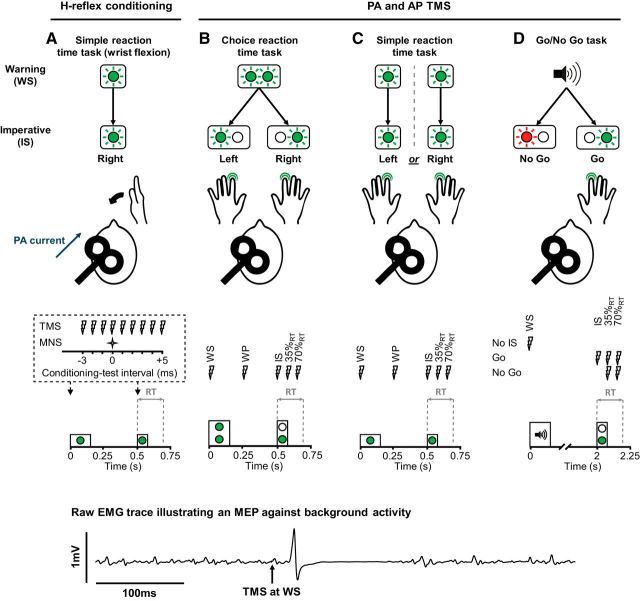
Reaction time tasks and stimulus timings. ***A***, For the SRTT in experiment 1, participants performed the task with their right wrist, and median nerve stimulus (MNS) and TMS stimulus timings were limited to WS and IS time points. ***B***, For the CRTT in experiment 2, a noninformative visual WS (left and right LEDs lit for 150 ms) preceded a left or right IS (75 ms duration), which cued a response with either the left or right index, respectively. ***C***, In experiment 3, participants performed separate blocks of the SRTT with their left and right index fingers. They received a visual WS (150 ms duration) before a visual IS (75 ms duration). ***D***, For the Go/No Go task in experiment 4, an auditory WS (500 Hz tone, 150 ms duration) preceded either a green (Go) or red (No Go) visual stimulus (75 ms duration), which cued the execution of a right index response and the withholding of a response, respectively. Within each experiment, stimuli were delivered at one of several time points in a trial: at the WS, in the warning period (WP) 0.25 s after the WS and before the IS (***B***, ***C***) at the IS and after the IS at 35% and 70% of the mean baseline reaction time (35%_RT_, 70%_RT_). TMS was delivered with the coil positioned to induce PA currents (see ***A***) only in experiment 1, and both PA and AP currents (position coil handle rotated 180° around the intersection of coil windings) in experiments 2–4. Note that for trials cueing a right-hand response, MEPs were recorded from the (right) responding hand; and for trials cueing a left-hand response, MEPs were recorded from the (right) nonresponding hand. An example raw EMG trace is shown at the bottom to illustrate the MEP against the background voluntary muscle activity during experiments 2–5.

Participants were seated 60 cm in front of colored (red or green) light-emitting diodes (LEDs) presented against a black background. They performed one of three different types of warned reaction time tasks: simple RT task (SRTT; Fig. 1*A* and 1*C*); choice RT task (CRTT; Fig. 1*B*); and Go/No Go task (Fig. 1*D*). In each of the tasks, a visual or auditory warning signal (WS) preceded a visual imperative signal (IS) by a fixed interval, and the latter signal cued a response. In experiment 1, participants were positioned with their right hand and wrist supported in an isometric dynamometer, with the shoulder in slight abduction, the elbow semiflexed and the forearm semipronated. They responded by attempting to flex the wrist “as quickly as possible.” In experiments 2–5, participants were positioned with their hands resting palm down on a table surface and the fingertips of the index fingers resting on a load cell. They responded by attempting to flex the index finger against the load cell “as quickly as possible.” Before the main experimental blocks in each task, all participants completed the following two blocks without TMS: a practice block followed by another block, which was used to estimate their mean baseline reaction time. Stimulus timings were controlled via Signal version 5.10 software (RRID:SCR_009601) connected to a data acquisition system (Power1401, CED).

#### Surface electromyogram

In experiment 1, surface electromyogram (EMG) electrodes (WhiteSensor 40713, Ambu) were placed 2 cm apart over the right flexor carpi radialis (FCR) muscle, with the ground positioned over the medial epicondyle of the humerus. In experiments 2–5, electrodes were placed in a belly tendon arrangement over the first dorsal interosseous (FDI) muscle of the left and right hand. The ground electrode was over the styloid process of the radius. Signals were amplified with a gain of 1000 (Digitimer), bandpass filtered (5–3000 Hz), digitized at 5 kHz (Power1401, CED), and analyzed with Signal version 5.10 software. EMG recordings enabled the measurement of reaction times and H-reflexes or motor evoked potentials (MEPs).

#### Transcranial magnetic stimulation

In experiment 1, a standard TMS device connected to a figure-of-eight coil (Magstim 200^2^, Magstim) was used to stimulate the FCR representation of the left primary motor cortex (M1). The coil was held tangentially on the scalp at an angle of 45° to the mid-sagittal plane to induce a posterior–anterior (PA) current across the central sulcus (Fig. 1*A*). The motor hot spot was found by searching for the position where slightly suprathreshold PA currents produced the largest and most consistent MEPs in FCR at rest. The position was marked on a cap worn by the participants. Resting motor threshold (RMT) with a PA current was defined as the lowest intensity to evoke an MEP of at least 0.05 mV in 5 of 10 consecutive trials while subjects were at rest. Thereafter, TMS was used to condition H-reflexes (van der Linden and Bruggeman, 1993; Niemann et al., 2017) rather than to elicit MEPs (see below). Stimulus intensity during the experiment was therefore below RMT (90% of RMT; i.e., at a level sufficient for evoking activity in the corticospinal tract, but producing only subthreshold depolarization of spinal motoneurons that can be detected by changes in H-reflex amplitude).

For experiments 2–5, MEPs in the dominant right FDI were evoked using a prototype controllable pulse parameter TMS device (cTMS3, Rogue Research Inc.; see also Peterchev et al., 2014) that was connected to a standard figure-of-eight coil (wing diameter, 70 mm; Magstim). The coil was held to induce either a PA current across the central sulcus (Fig. 1*A*) or an oppositely directed anterior—posterior (AP) current, whereby the position of the coil handle was reversed around the intersection of coil windings (Sakai et al., 1997). PA and AP currents tend to activate the corticospinal tract via different sets of excitatory synaptic inputs (Di Lazzaro et al., 2001; see below). Here, we used the following different pulse durations for PA and AP current directions: long-duration (120 μs) pulses in the PA direction; and short-duration (30 μs) pulses in the AP direction. It was recently shown that these combinations of current direction and pulse duration achieve the greatest distinction in the recruitment of these distinct synaptic inputs (D'Ostilio et al., 2016; Hannah and Rothwell, 2017).

The motor hot spot for the FDI was defined in a manner similar to that for the FCR. The active motor threshold (AMT) with PA and AP currents was defined as the lowest intensity to evoke a discernible MEP in 5 of 10 consecutive trials, while subjects maintained slight voluntary contraction (5–10% of maximum voluntary EMG amplitude during isometric finger flexion). Stimulation intensity during experiments 2–5 was set to that which produced a mean MEP amplitude of ∼1 mV (A_1mV_) during slight voluntary contraction (5–10% maximum voluntary EMG amplitude) for each of the PA and AP currents.

#### Peripheral nerve stimulation

In experiment 1, square wave pulses (1 ms pulses) were delivered to the median nerve just proximal to the elbow via cup electrodes (cathode proximal), which were connected to a constant-current stimulator (DS7A, Digitimer). Initially, stimulus intensity was gradually increased to obtain maximal H-reflex and M-wave responses in the FCR. Then the stimulus intensity was set to evoke H-reflexes with an amplitude of >5% of maximal M-wave amplitude (Pierrot-Deseilligny and Burke, 2012). Mean unconditioned H-reflex amplitudes at the warning and imperative signals were 17 ± 3% and 16 ± 3% of maximal M-wave amplitude, respectively.

#### Experimental design: assessing excitatory synaptic inputs to corticospinal neurons with H-reflex conditioning

A single TMS pulse can activate separate excitatory synaptic inputs to the corticospinal neurons, which arrive at different latencies and produce temporally distinct discharges in the pyramidal tract (I-waves; Kaneko et al., 1996; Di Lazzaro et al., 1998). We used a method of conditioning the H-reflex with TMS to test for selective suppression of the inputs responsible for early and late I-wave discharges (van der Linden and Bruggeman, 1993; Niemann et al., 2017) during the preparatory period of a simple reaction time task. The rationale for the paradigm is that TMS-evoked I-waves descending the corticospinal tract will produce EPSPs at the spinal motoneurons. The TMS intensity is set below RMT so that the I-waves produce only subliminal depolarization of the spinal motoneurons, which increases the probability of them firing in response to another excitatory input. Thus, if an Ia afferent volley arrives at the same time or shortly after the TMS-evoked corticospinal volleys, the resulting H-reflex will be facilitated compared with control H-reflexes where no TMS is delivered (van der Linden and Bruggeman, 1993; Niemann et al., 2017). Similarly, if the interval between the conditioning TMS stimulus and test H-reflex stimulus is altered so that the afferent volley reaches the spinal motoneurons before the TMS volleys arrive, the H-reflex will be unaffected since the efferent response will already have been generated. The interval between the conditioning TMS stimulus and the test H-reflex stimulus that produced coincident arrival of the corticospinal and afferent volleys at the spinal motoneurons, and thus facilitated the H-reflex, can be considered to be 0 ms (i.e., there is zero delay between their arrivals). Positive values for the afferent–corticospinal volley delay (e.g., +1 ms) then reflect the delayed arrival of the afferent compared with corticospinal volleys, while negative values (e.g., −1 ms) reflect the earlier arrival of the afferent volleys compared with the corticospinal volleys.

It is important to note that the time of arrival of the early and late I-waves at the spinal motoneurons differs by several milliseconds (Day et al., 1989a; Sakai et al., 1997; Di Lazzaro et al., 1998), thus their contribution to the period of H-reflex facilitation can be partly dissociated by using different conditioning test intervals. Facilitation at intervals resulting from near-coincident arrival of the first corticospinal volleys (early I-waves) and afferent volleys (e.g., 0 and +1 ms) should correspond to EPSPs generated by those same early I-waves, while facilitation at longer intervals (e.g., +3, +4, and +5 ms) should receive an important contribution from EPSPs generated by I-waves arriving later. Consequently, changes in the level of H-reflex facilitation at different conditioning test intervals throughout the premovement period (i.e., from the warning to the imperative signal) would, all other things being equal, be expected to reflect changes in I-wave composition. For example, greater facilitation at 0 ms and reduced facilitation at +4 ms would reflect an increased presence of early I-waves and a reduced presence of late I-waves, respectively. The dynamical systems approach posits that during movement preparation there is an overall balance of suppression and facilitation of inputs to corticospinal neurons. However, it seems unlikely that inhibition and facilitation would be equally distributed to early and late I-wave inputs. We therefore proposed that the early (early I-waves) and later period of H-reflex facilitation (late I-waves) would be differentially, and potentially oppositely, affected at the time of the imperative by comparison with the warning signal.

##### Experiment 1: simple reaction time task with H-reflex conditioning.

We studied reflexes in the FCR because it can be difficult to reliably evoke H-reflexes in hand muscles (Mazzocchio et al., 1995). Single median nerve stimulation pulses were used to evoke test H-reflexes in the right FCR muscle in separate trials at either the time of the warning or the imperative signal. In some trials, a conditioning stimulus consisting of subthreshold TMS of the left M1 was delivered at different times relative to the median nerve stimulus, from 3 ms before to 5 ms after in 1 ms increments. Note that the earliest facilitation of the H-reflex, resulting from coincidental arrival of corticospinal and afferent volleys (0 ms as mentioned above), typically occurs when the TMS follows the peripheral nerve stimulus by 3 ms because of the faster conduction to the spinal motoneurons in the corticospinal pathway compared with the peripheral afferent pathway. The experiment was performed at rest (i.e., no background muscle contraction) and with the application of near-threshold PA currents, which we presumed would recruit a mixture of early and late I-waves (Day et al., 1989a; Di Lazzaro et al., 1998).

Eleven individuals participated in the experiment. The main experiment consisted of two blocks of 122 trials (244 trials in total) of right wrist flexor responses. Unconditioned control H-reflexes were evoked in the right FCR at the warning and at the imperative signal (20 and 20 trials in total, respectively). Ten trials were included for each conditioning test interval of the conditioned H-reflexes (180 trials in total), and 24 catch trials with no stimulation or imperative signal were also included. Trial order was randomized, and the intertrial interval was set to 8 s. A 5 min rest period separated each block.

#### Experimental design: assessing excitatory synaptic inputs to corticospinal neurons with directional TMS

Many factors can contribute to the time course of H-reflex facilitation produced by a subthreshold TMS pulse. The initial millisecond or so is probably dominated by the interaction between monosynaptic inputs from the fastest corticospinal and Ia afferent pathways. Thereafter, in addition to the arrival of late corticospinal I-waves, there can be contributions from slower conducting fibers, Ib afferents activated by the H-reflex stimulus, presynaptic effects, and indirect inputs from cortex coming via propriospinal, reticulospinal, or even segmental interneuronal pathways. Changes in the contribution from any of these pathways in the preparation for movement could contribute to the results in experiment 1, although they would not easily account for the specificity of the timing. Thus, to provide more support for our hypothesis that these effects were likely to be related to the suppression of late I-wave inputs, we added a second series of experiments using directional effects of TMS.

These experiments investigated differential changes in the amplitudes of PA- and AP-evoked MEPs during movement preparation. PA and AP currents recruit different proportions of early and late I-waves, and thus comparing the relative changes in MEP amplitudes can help reveal differential changes in the activity of different I-waves (Hanajima et al., 1998; Hannah and Rothwell, 2017). Practically, this method also allowed us to more fully investigate the time course of changes in cortical excitability during movement preparation by including a greater number of stimulus time points. In each experiment, single-pulse TMS was delivered over the FDI representation of the left motor cortex in separate trials and, at various times, to evoke MEPs in the right FDI muscle.

Experiments 2–5 were performed with slight background muscle contraction, ensuring that MEPs could be evoked by low-intensity stimulation. This was necessary because differences in MEP latencies between PA and AP currents are obscured at higher intensities since pulses then recruit a mixture of I-waves (Day et al., 1989a; Sakai et al., 1997; Di Lazzaro et al., 2001). Participants received intermittent verbal feedback regarding voluntary rms EMG amplitude (target 5–10% maximum) to ensure they maintained a consistent level of voluntary muscle activity throughout the tasks by lightly flexing the index fingers against the load cell. Feedback was given in between trials in relation to the action that was required (increase or decrease activity) and the hand it related to (left, right, or both), and only when activity was consistently outside the bounds for three or more consecutive trials.

##### Experiment 2: choice reaction time task with directional TMS.

Previous studies adopting a CRTT in which an uninformative WS precedes an informative IS reported a suppression of MEPs in all response-relevant muscles toward the time of the IS (Touge et al., 1998; Duque and Ivry, 2009), for example, in both left-hand and right-hand muscles. The present experiment served two purposes. The first purpose was to confirm the data from the previous experiment by showing that late I-waves (AP MEPs) in the eventual responding hand are suppressed more than early I-waves (PA MEPs) in the preparatory period. The second purpose was to extend these results and to ask whether the same is true in the other potential respondent muscle (i.e., the nonresponding hand; Fig. 1*B*).

Fifteen individuals participated in the experiment. The main experiment consisted of eight blocks, with TMS delivered in the four blocks with a PA current and four blocks with an AP current. The order of blocks alternated between PA and AP, and the first block was randomly assigned to either the PA or AP direction. Each block consisted of 50 trials: 25 each of left and right index cues. Each combination of response hand and TMS timing was repeated 5 times per block, and therefore 20 times over the course of four blocks each for PA and AP currents, resulting in 20 MEPs per time point for each current direction and response cue. The order of trials was pseudorandomized across the 10 different combinations of response cue and TMS timing, and the intertrial interval was set to 5 ± 0.5 s. A 5 min rest period separated each block.

##### Experiment 3: simple reaction time task with directional TMS.

Preparatory inhibition of MEPs has been reported in the responding effector during warned SRTTs toward the time of the imperative signal (Hasbroucq et al., 1997; Touge et al., 1998; Greenhouse et al., 2015). Surprisingly, preparatory inhibition of MEPs has also been reported in “response-irrelevant” muscles, for example, a homologous or nonhomologous muscle on the contralateral side of the body that is not a response option (Greenhouse et al., 2015). Preparatory inhibition here, where it may be desirable to fully suppress the output neurons of the response-irrelevant muscle representation, might be enacted through a less selective mechanism (e.g., somatic inhibition of corticospinal output neurons that could resemble the sort of gating mechanism implied by the subthreshold hypothesis). This would be expected to suppress the response to all excitatory I-wave inputs and might, therefore, affect PA and AP MEPs similarly. We compared preparatory motor inhibition in the absence of choice between response options (i.e., where there is only one response option) and when the muscle representation was or was not a potential response option.

Thirteen individuals participated in the experiment. The main experiment consisted of four blocks (Fig. 1*C*), two blocks with each hand and with TMS delivered in one block with a PA current and the other with an AP current. The order of blocks alternated between PA and AP. Each block consisted of only right or left index responses, and participants were told before each block which hand they were required to respond with. Blocks consisted of 120 trials. In two blocks, MEPs were evoked in the right hand when it was the responding (response-relevant) hand, and in the other two blocks MEPs were evoked in the right hand when it was the nonresponding (response-irrelevant) hand (i.e., when a left-hand response was required). To prevent anticipation of the IS and premature responses, catch trials (20 in total for PA and AP conditions) were included where a warning appeared, but no imperative signal was presented and no TMS was delivered, and participants were instructed not to respond on these trials. This design resulted in 20 MEPs per time point for each current direction and response hand. The order of trials within each block was pseudorandomized across the five different TMS timings, and the intertrial interval was set to 5 ± 0.5 s. A 2 min break was given after the first 50 trials of each block, and a 5 min rest period separated each block to prevent fatigue due to the sustained voluntary muscle contraction.

##### Experiment 4: Go/No Go task with directional TMS.

Several studies have reported that during successful outright suppression of a response in reaction to a sudden Stop or No Go signal involves a broad “global” inhibition of response-relevant and response-irrelevant muscle representations after the IS, at around the time when a volitional muscle activity would be otherwise have been expected (Hoshiyama et al., 1997; Badry et al., 2009; Greenhouse et al., 2015). We hypothesized that successful stopping in a Go/No Go task would involve direct inhibition (e.g., somatic inhibition) of corticospinal output neurons and be reflected by a similar suppression of both PA- and AP-evoked MEPs.

Twelve individuals participated in the experiment. The main experiment consisted of eight blocks (Fig. 1*D*), with TMS delivered in the four blocks with a PA current and four blocks with an AP current, and the order of blocks alternating between PA and AP. Since any preparatory inhibition before the imperative might confound attempts to explore subsequent inhibition after this time, we attempted to minimize any preparatory inhibition by increasing the interval between the warning and imperative to 2 s (Touge et al., 1998). We also used an auditory warning in the present experiment to ensure that it was unambiguous and distinct from the two possible visual imperative signals.

In total there were 70 trials per block. Trials included the following: TMS-alone trials delivered at the time of the WS, although without the presentation of the WS or IS (10/block); Go trials with no TMS (10/block); Go trials with TMS at the IS (12/block), 35% of RT (35%_RT_; 12/block), and 70%_RT_ (12/block); as well as No Go trials with TMS at 35%_RT_ (7/block) and 70%_RT_ (7/block). Thus, blocks consisted of 10 trials with TMS at the WS, serving as the baseline measure of corticospinal excitability, along with 46 Go trials and 14 No Go trials, which resulted in a Go/No Go ratio of 3.3:1. Four blocks were performed for each TMS current direction to ensure an adequate number of MEPs at each time point for the No Go trials (24 each). The order of trials within each block was pseudorandomized across the seven different types of trials, and the intertrial interval was set to 5 ± 0.5 s.

##### Experiment 5: relationship of reaction times and trial-by-trial variability in MEPs assessed with AP TMS.

Following on from the previous experiments, we wanted to test the validity of the assumption that the preparatory inhibition reflected a mechanism for preventing movement during preparation. We hypothesized that if individuals do use such a mechanism, then it should be observable on a trial-by-trial basis: trials with greater suppression of MEPs would be associated with extended reaction times. Suprathreshold TMS around the time of the imperative signal can potentially delay contralateral responses (Day et al., 1989b) and impair detection of EMG-derived reaction time because of the silent period following the MEP in preactivated muscles. We therefore used a bilateral response version of the SRTT (Fig. 1*C*) so that reaction times on the side ipsilateral to the TMS (left-hand side) could be used as a surrogate of the actual reaction time on the contralateral (right-hand) side (Schneider et al., 2004).

Eleven individuals participated in the experiment. They performed an initial familiarization consisting of 20 trials without TMS, followed by a further 60 practice trials (55 response trials and 5 catch trials in total) to obtain stable reaction times. The main experiment consisted of three blocks of the SRTT with AP TMS delivered in each. Blocks consisted of 112 trials (336 trials in total) of simultaneous right and left index responses. MEPs were evoked in the right hand at the time of the warning signal (120 trials in total) and at the imperative signal (120 trials in total), since the latter was most often associated with the greatest preparatory MEP suppression (experiments 2–3). Catch trials (36 trials in total) and trials without TMS (60 trials in total) were included as before. Trial order was pseudorandomized across the four different trial types, and the intertrial interval was set to 5 ± 0.5 s. A 2 min break was given after the first 66 trials of each block, and a 5 min rest period separated each block.

#### Data analysis

EMG data were analyzed off-line using Signal version 5.10. For experiment 1, two dependent variables were measured on a trial-by-trial basis and were used to create a mean value for each time point (WS and IS) and conditioning test interval, as follows: (1) H-reflex peak-to-peak amplitude; and (2) reaction time measured from the onset of the IS to the onset of volitional muscle activity.

For experiments 2–5, four dependent variables were measured on a trial-by-trial basis and were used to create a mean value for each response hand (responding vs nonresponding, experiments 2 and 3), current direction, time point of TMS, and trial type (Go and No Go, experiment 3), as follows: (1) MEP peak-to-peak amplitude; (2) MEP onset latency measured from the time of TMS pulse delivery to the onset of the MEP; (3) voluntary rms EMG amplitude over the 100 ms before the TMS pulse; and (4) reaction time measured as above. The onset of volitional muscle activity was defined as an increase in the rms EMG (5 ms time constant) amplitude that exceeded the pre-TMS rms EMG (100 ms) by ≥2 SDs for at least 10 ms. The onset of MEPs was determined visually from the raw EMG traces (Day et al., 1989a; Hamada et al., 2013). MEP latencies were measured for both current directions and at all TMS time points for experiment 2 to verify that any differences between current directions persisted throughout the task. In experiments 3 and 4, MEP latencies were measured for each current direction only at the earliest TMS time point (WS). Measurement of the voluntary rms EMG amplitude 100 ms before each TMS pulse enabled comparison of the level of volitional muscle activity across different current directions and TMS pulse timings to ensure that any differences in the amplitudes of MEPs were not confounded by differences in volitional muscle activity.

In experiment 1, trials were included for analysis if they met the following criteria: (1) RT was >80 ms and within 3 SDs of the mean; and (2) rms EMG in the 100 ms before the IS was within ±2 SDs of the mean for that block. For experiments 2–5, trials were included for further analysis if they met the following criteria: (1) RT was >80 ms and within 3 SDs of the mean; (2) response was correct (e.g., left index response only for trials with left cues or no response in No Go trials); and (3) voluntary rms EMG before the TMS pulse was within ±2 SDs of the mean for that block. The average numbers of trials removed per individual in each experiment were as follows: 6%, experiment 1; 7%, experiment 2; 9%, experiment 3; 6%, experiment 4 (4% in Go trials vs 15% in No Go trials); and 22% of IS trials in experiment 4 leaving 94 ± 5 trials for analysis.

#### Statistical analyses

Data are reported as the group mean ± SEM. Repeated-measures ANOVA (rmANOVA) was used to evaluate the majority of the data, with Bonferroni-corrected, repeated-measures *t* tests used to follow up significant main effects or interactions. *p* values <0.05 were considered to be significant. Where necessary, the Greenhouse–Geisser procedure was applied to correct for violations of sphericity in ANOVA.

##### Experiment 1: SRTT with H-reflex conditioning.

Data were assessed to identify the first conditioning-test interval at the WS time point where the mean conditioned H-reflex amplitude exceeded the mean unconditioned H-reflex amplitude by at least 2 SEMs of all 20 unconditioned trials. Conditioning-test intervals were then realigned on an individual basis such that this interval (afferent–corticospinal volley delay) corresponded to 0 ms, reflecting presumed coincident arrival of the afferent and corticospinal volleys at the spinal motoneurons (i.e., zero delay between their arrivals) as described earlier. Because of the different onsets of facilitation across individuals, analyses were limited to the unconditioned response and conditioned responses at realigned intervals between −1 to +5 ms.

Two-way rmANOVA was used to determine the effects of time point (WS, IS) and afferent–corticospinal volley delay (unconditioned, −1, 0, 1, 2, 3, 4, 5) on absolute H-reflex amplitudes and RTs. For *post hoc* analyses assessing the effect of afferent–corticospinal volley delay on the H-reflex, *t* tests were performed on absolute conditioned H-reflexes by comparing them to the unconditioned H-reflex at the same time point, which served as the baseline measure of spinal motoneuron excitability. When comparing H-reflexes across different stimulation time points for a given afferent–corticospinal volley delay, data at each delay were normalized at each time point by expressing the mean conditioned H-reflex amplitude relative to the mean unconditioned H-reflex amplitude. This controlled for potential differences in baseline H-reflex amplitude at the WS and IS. Paired *t* tests were performed on the normalized data.

##### Experiment 2: CRTT with directional TMS.

Three-way rmANOVA was used to determine the effects of hand (right hand responding, right hand nonresponding), current direction (PA, AP), and time of TMS [WS, warning period (WP), IS, 35%_RT_, 70%_RT_] on absolute MEP amplitudes, MEP latencies, voluntary rms EMG amplitude, and RTs. For *post hoc* analyses assessing the effects of time point on MEPs within a particular response hand and current direction, *t* tests were performed on absolute MEPs by comparing them to those at the WS, which served as the baseline measure of corticospinal excitability. When comparing current directions at each time point for a given hand, data at each time point were normalized by expressing the mean MEP size as a ratio relative to the mean MEP size at the WS, to control for potential differences in baseline MEP amplitude, and paired *t* tests were performed on the normalized data.

##### Experiment 3: SRTT with directional TMS.

Data were analyzed in a manner similar to that in experiment 2, whereby three-way rmANOVA was used to determine the effects of hand (right hand responding, right hand nonresponding), current direction (PA, AP), and time of TMS (WS, WP, IS, 35%_RT_, 70%_RT_) on absolute MEP amplitudes, voluntary rms EMG amplitude, and RTs. However, since MEP latencies were measured only at the WS time point, a two-way rmANOVA was used to determine the effects of hand (right hand responding, right hand nonresponding) and current direction (PA, AP).

##### Experiment 4: Go/No Go task with directional TMS.

We analyzed the data in two stages. First, we wanted to test for the presence of preparatory suppression of MEPs at the IS and to examine whether this was different for PA and AP current directions. Two-way rmANOVA was used to assess the effects of current direction (PA, AP) and time (WS, IS) on absolute MEP amplitudes. For the second analysis, we were particularly interested in whether the suppression of MEPs after the IS in the No Go condition was different between AP and PA currents. To minimize any bias introduced by potential preparatory suppression of MEPs at the IS, we chose to normalize the amplitude of MEPs at 35%_RT_ and 70%_RT_ to those at the IS and did this for both Go and No Go trials. Three-way rmANOVA was used to examine the effects of trial type (Go, No Go), current direction (PA, AP), and time (35%_RT_, 70%_RT_) on normalized MEP amplitudes. For *post hoc* analyses assessing the effects of time on MEPs within a trial type and current direction, *t* tests were performed on absolute MEPs by comparing them to those at the IS. When comparing current directions at each time for a trial type, paired *t* tests were performed on the data for normalized MEP amplitudes. Voluntary rms EMG data were analyzed in the same manner as MEPs. MEP latencies were measured only at the time of the WS, and thus a paired *t* test was performed to compare them for PA and AP currents. A two-way rmANOVA was used to evaluate the effects of current direction (PA, AP) and time (Go alone, IS, 35%_RT_, 70%_RT_) on RTs in Go trials.

##### Experiment 5: relationship of reaction times and trial-by-trial variability in MEPs assessed with AP TMS.

For each individual, right-hand (i.e., responding hand) MEP amplitudes during IS trials and WS trials were first normalized to the EMG amplitude preceding the TMS pulse in each trial to account for variations in background muscle activity. Normalized MEP amplitudes from IS trials were then each expressed as a percentage change relative to the average amplitude of normalized MEPs from the WS trials. Left-hand reaction times from IS trials were ranked within each individual, expressed as a percentage of the total number of trials and then binned according to each consecutive 10 percentile window (i.e., 0 to 10th, 10th to 20th … 90th to 100th, in which the 0 to 10th percentile would contain the fastest 10% of reaction times). The corresponding average MEP amplitude changes from the right hand were plotted as a function of reaction time percentile bins, and Pearson bivariate correlations were used to assess the relationship between them at both the individual and group average level.

## Results

### Thresholds and baseline response amplitudes

The resting motor threshold in experiment 1 was 55 ± 5% maximum stimulator output, such that the 90% RMT conditioning stimulus was 50 ± 5% of maximum stimulator output. Motor thresholds measured at the start of experiments 2–5 and absolute MEP amplitudes measured at the control TMS time point (WS) in each experiment are shown in Table 1. AP pulses required much greater stimulus intensities than PA currents (all *p* < 0.001). This was to be expected given the following: (1) thresholds are greater for AP pulses even when similar pulse durations are applied (D'Ostilio et al., 2016; Hannah and Rothwell, 2017); and (2) the strength–duration behaviors of PA- and AP-sensitive inputs are different (D'Ostilio et al., 2016). The level of background muscle activity, quantified as the root mean square (rms) amplitude, was typically ∼0.05 mV during experiments 2–5.

**Table 1.** Motor thresholds and baseline response amplitudes for experiments 2–5

	Experiment 2: CRTT (*n* = 15)		Experiment 3: SRTT (*n* = 13)		Experiment 4: Go/No Go (*n* = 12)		Experiment 5: bilateral SRTT (*n* = 11)
	PA	AP	PA	AP	PA	AP	AP
AMT (%MSO)	26 ± 1	78 ± 2	27 ± 1	76 ± 1	27 ± 2	74 ± 2	74 ± 2
A_1mV_ (%MSO)	31 ± 1	89 ± 2	32 ± 1	91 ± 1	32 ± 2	85 ± 2	92 ± 2
A_1mV_/AMT (%)	117 ± 1	115 ± 1	123 ± 2	121 ± 2	119 ± 2	116 ± 1	125 ± 3
MEP amplitude at WS (mV)	1.2 ± 0.1	1.2 ± 0.1	1.2 ± 0.1 (R)	1.2 ± 0.1 (R)	1.2 ± 0.1	1.3 ± 0.1	1.2 ± 0.1
			1.1 ± 0.1 (NR)	1.2 ± 0.1 (NR)			

Data are reported as the mean ± SEM. NR, nonresponding; %MSO, percentage of maximum stimulator output; R, responding.

### Experiment 1: SRTT with H-reflex conditioning

#### H-reflex amplitude

**Figure 2.**
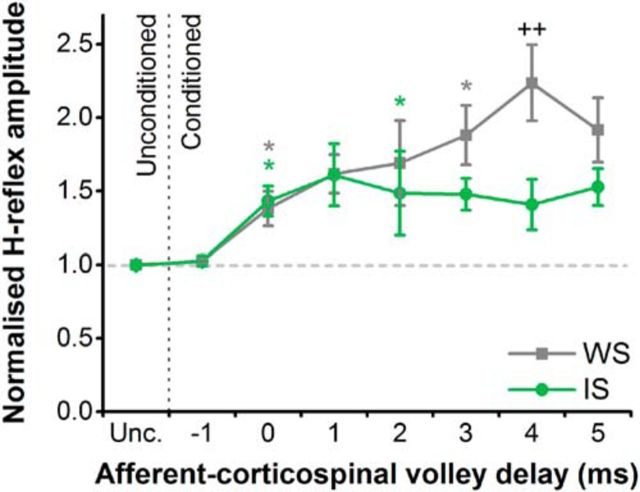
H-reflexes conditioned with TMS during the simple reaction time task. The interval between the conditioning TMS stimulus and the test H-reflex stimulus that produced coincident arrival of the corticospinal and afferent volleys at the spinal motoneurons, and thus facilitated the H-reflex, was considered to be 0 ms (i.e., the afferent–corticospinal volley delay is 0). Positive values for the delay (e.g., +1 ms) then reflected delayed arrival of the afferent compared with corticospinal volleys, while negative values (e.g., −1 ms) reflected the earlier arrival of the afferent volleys compared with the corticospinal volleys. During the simple reaction time task, H-reflexes in the FCR muscle were facilitated to a lesser extent at the IS than the WS, specifically when the arrival of the afferent volleys at the spinal motoneurons was delayed relative to the corticospinal volleys (4 ms). By contrast, H-reflexes were facilitated to a similar extent at the IS and WS when the afferent and corticospinal volleys arrived coincidentally at the spinal motoneurons (0 ms). *p < 0.05, compared with unconditioned (Unc.) H-reflex within each time point (WS and IS); ++*p* < 0.01, IS vs WS.

At afferent–corticospinal volley delays corresponding to the earliest facilitation of the H-reflex by TMS, there was no change in the level of facilitation during the preparatory period. However, at delays corresponding to the later periods of H-reflex facilitation, there was a decrease in the level of facilitation during the preparatory period (Fig. 2).

There was no difference in the amplitude of the unconditioned H-reflex at the time of the WS compared with that at the IS (1.10 ± 0.41 vs 1.03 ± 0. 36 mV; *t*_(10)_ = 1.382, *p* = 0.197). The statistics showed a significant time × afferent–corticospinal volley delay interaction (*F*_(7,70)_ = 5.881, *p* < 0.001). Subsequent paired *t* tests revealed a smaller conditioned H-reflex amplitude for the IS versus the WS time point at a 4 ms delay, although comparisons at 2 and 3 ms delays did not survive the Bonferroni correction. Comparison of conditioned H-reflex amplitudes with respect to unconditioned H-reflex amplitudes at each time point indicated that responses were significantly facilitated at 0 ms at both the WS and IS time points, and at 3 ms for the WS and 2 ms for the IS time points. The remaining intervals did not survive the Bonferroni correction.

#### Reaction time

Reactions times for the unconditioned H-reflex condition were 181 ± 6 and 179 ± 6 ms when stimuli were delivered at the WS and IS, respectively. rmANOVA showed no main effect of time (*F*_(1,10)_ = 1.121, *p* = 0.315) or afferent–corticospinal volley delay (*F*_(7,70)_ = 1.441, *p* = 0.203), and no time × afferent–corticospinal volley delay interaction (*F*_(3.228,32.284)_ = 1.037, *p* = 0.393).

Many descending and afferent pathways could potentially contribute to the time course of H-reflex facilitation produced by a subthreshold TMS pulse, and changes in any of their contributions could thus influence the results in experiment 1. We therefore attempted to verify that these results were specifically related to the suppression of late I-wave inputs by adding a second series of experiments using the directional effects of TMS.

### Experiment 2: CRTT with directional TMS

#### MEP amplitude

**Figure 3.**
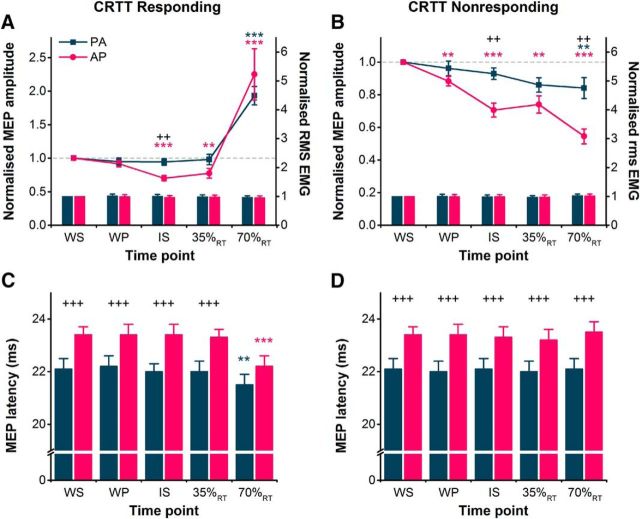
***A***, ***B***, During the choice reaction time task, MEP amplitudes in the right FDI shown normalized to the WS time point (colored lines, left *y*-axis) were suppressed more for AP currents than for PA currents at the IS during right hand-responding trials (***A***) and at the IS and 70%_RT_ in right-hand nonresponding trials (***B***). The facilitation of MEPs in right hand-responding trials at 70%_RT_ was similar for both current directions (***A***). Voluntary rms EMG (colored bars, right *y*-axis) measured before the TMS pulses is shown normalized to values at the WS, and was similar for PA and AP currents across different time points for right hand-responding (***A***) and nonresponding trials (***B***). ***C***, ***D***, MEP latencies were longer for AP currents compared with PA currents in both right hand-responding (***C***) and nonresponding (***D***) trials at all time points except 70%_RT_ in responding trials. **p < 0.01, ***p < 0.001, compared with the WS time point within each current direction; ++*p* < 0.01, +++*p* < 0.001, AP vs PA.

MEPs evoked by AP pulses were suppressed to a greater extent than PA-evoked MEPs during the preparatory period of a choice reaction time task, both when the right hand was the eventual responding hand and nonresponding hand (Fig. 3*A*,*B*). The facilitation of MEPs in the right hand immediately before movement was similar for PA and AP MEPs. This was supported by a significant hand × current direction × time interaction in the rmANOVA (Table 2). Subsequent paired *t* tests for right-hand responses revealed that AP-evoked MEPs, but not PA MEPs, were suppressed at the time of the IS and 35%_RT_ compared with those at the WS, but both PA and AP MEPs were facilitated just before volitional EMG onset at 70%_RT_ (Fig. 3*A*). Additionally, comparison of normalized MEP amplitudes indicated a greater suppression of AP MEPs compared with PA MEPs at the time of the IS (Fig. 3*A*). When the right hand was the nonresponding hand, paired *t* tests revealed that AP-evoked MEPs were suppressed at all time points compared with the WS, whereas PA MEPs were suppressed only at 70%_RT_ (Fig. 3*B*). Furthermore, the suppression of AP-evoked MEPs (normalized to WS) was greater than that of PA-evoked MEPs at the time of the IS and at 70%_RT_.

**Table 2.** Results of rmANOVAs conducted for experiments 2 and 3

	Experiment 2: CRTT (*n* = 15)		Experiment 3: SRTT (*n* = 13)	
	*F*_[df,error]_	*p*	*F*_[df,error]_	*p*
MEP amplitude				
Hand	1.114_[1,14]_	0.178	16.551_[1,12]_	0.002
Current direction	30.133_[1,14]_	<0.001	0.222_[1,12]_	0.646
Time	21.389_[1.89,26.532]_	<0.001	17.337_[1.86,22.274]_	<0.001
Hand × current direction	2.417_[1,14]_	0.142	0.256_[1,12]_	0.616
Hand × time	34.991_[4,56]_	<0.001	27.486_[2.041,24.487]_	<0.001
Current direction × time	3.170_[4,56]_	0.020	0.656_[2.11,25.275]_	0.535
Hand × current direction × time	4.609_[1.69,56]_	0.025	2.930_[4,48]_	0.015
MEP latency				
Hand	7.959_[1,14]_	0.014	5.212_[1,12]_	0.041
Current direction	51.152_[1,14]_	<0.001	41.485_[1,12]_	<0.001
Time	9.723_[2.52,35.295]_	<0.001		
Hand × current direction	1.513_[1,14]_	0.239	1.706_[1,12]_	0.216
Hand × time	18.292_[4,56]_	<0.001		
Current direction × time	1.131_[4,56]_	0.351		
Hand × current direction × time	3.126_[2.39,56]_	0.049		
Voluntary rms EMG amplitude				
Hand	4.325_[1,14]_	0.056	3.483_[1,12]_	0.087
Current direction	0.018_[1,14]_	0.895	0.131_[1,12]_	0.723
Time	0.325_[1.834,25.682]_	0.059	2.596_[2.248,26.981]_	0.087
Hand × current direction	2.418_[1,14]_	0.142	0.016_[1,12]_	0.900
Hand × time	4.512_[4,56]_	0.026	0.782_[2.298,27.575]_	0.483
Current direction × time	1.757_[4,56]_	0.150	0.778_[4,48]_	0.545
Hand × current direction × time	1.424_[4,56]_	0.238	0.864_[2.32,27.838]_	0.447
Reaction time				
Hand	4.727_[1,14]_	0.047	4.593_[1,12]_	0.053
Current direction	0.002_[1,14]_	0.963	3.807_[1,12]_	0.075
Time	22.292_[1.981,27.737]_	<0.001	74.832_[4,48]_	<0.001
Hand × current direction	0.047_[1,14]_	0.831	0.389_[1,12]_	0.545
Hand × time	6.284_[4,56]_	<0.001	9.461_[4,48]_	<0.001
Current direction × time	0.726_[4,56]_	0.578	1.802_[4,48]_	0.144
Hand × current direction × time	0.660_[4,56]_	0.622	1.216_[4,48]_	0.317

#### MEP latency

The latency of AP-evoked MEPs was greater than that of PA-evoked MEPs for right hand-responding and nonresponding trials at nearly all time points (Fig. 3*C*,*D*). In the statistics, rmANOVA revealed an interaction of hand × current direction × time (Table 2). Subsequent paired *t* tests suggested this was driven by the generally greater latency of AP versus PA MEPs except when evoked during right-hand responses at 70%_RT_ (Fig. 3*C*), where both AP and PA MEPs were strongly facilitated (Fig. 3*A*). This confirms that we achieved selective recruitment of AP and PA inputs through the majority of the task, especially at the time when preparatory inhibition was observed.

#### Voluntary rms EMG amplitude

The voluntary rms EMG amplitude in the right hand was generally consistent across current directions, right hand-responding and nonresponding trials, and time points (Fig. 3*A*,*B*), as indicated by a general lack of main effects and interactions in the rmANOVA (Table 2). Although an interaction of hand × time was suggestive of a small decrease in voluntary rms EMG amplitude at 70%_RT_ for right hand-responding trials versus nonresponding trials, regardless of current direction, a paired *t* test on the pooled EMG amplitudes of AP and PA conditions revealed no significant difference between responding and nonresponding trials (*p* = 0.139). Thus, the differences observed between AP and PA pulses in MEP amplitudes and latencies are unlikely to have been confounded by potential differences in the level of voluntary muscle activity.

#### Reaction time

As expected from previous work (Pascual-Leone et al., 1992), reactions times were shortened for right hand-responding and nonresponding trials (i.e., left-hand responses), regardless of current direction, when TMS was delivered around the time of the IS, consistent with an effect of intersensory facilitation (Nickerson, 1973). Additionally, reaction times were increased for right hand-responding trials when delivered at 70%_RT_ (see Fig. 6). This was supported by a significant interaction of hand × time in the rmANOVA (Table 2). This may relate to the silent period that follows the MEP in contracting muscle (Day et al., 1989b). There was no effect of current direction or any interactions with current direction. Follow-up paired *t* tests showed that, when collapsed across current directions, reaction times were shortened when TMS was delivered at the IS and 35%_RT_ compared with at the WS for right hand-responding trials (both *p* ≤ 0.002) and at the IS for nonresponding trials (*p* < 0.001), and lengthened when delivered at 70%_RT_ during right-hand responses (*p* = 0.001; see Fig. 6).

### Experiment 3: SRTT with directional TMS

#### MEP amplitude

**Figure 4.**
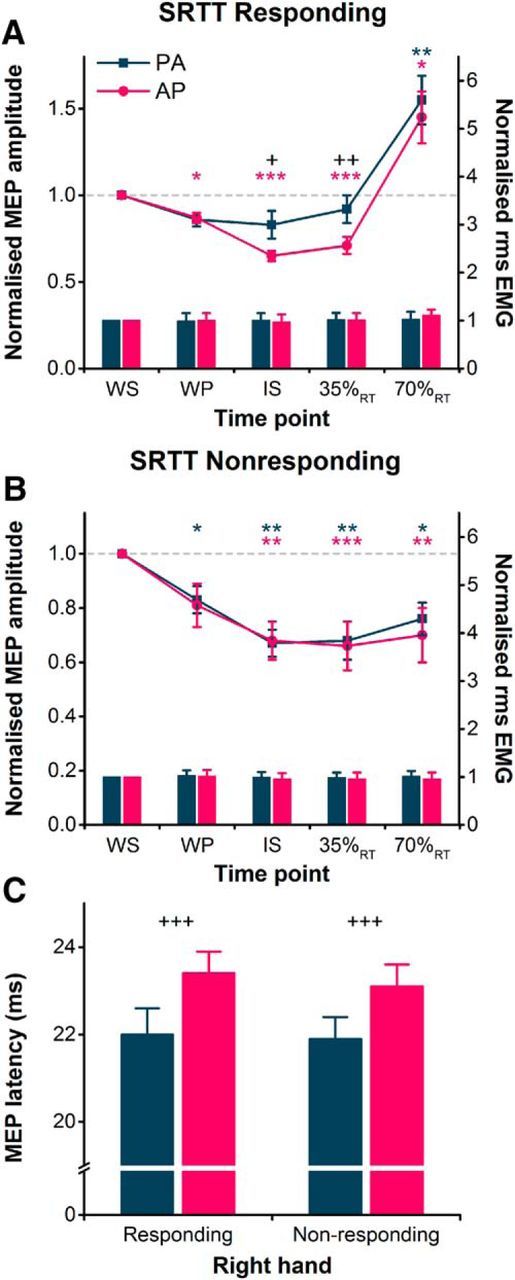
***A***, During the simple reaction time task, MEP amplitudes in the right FDI shown normalized to the WS time point (colored lines, left *y*-axis) and were suppressed more for AP currents than for PA currents at the IS and 35%_RT_ during right hand-responding blocks. The facilitation of MEPs in the same block at 70%_RT_ was similar for both current directions. ***B***, However, for right-hand nonresponding blocks, normalized MEP amplitudes were suppressed to a similar extent for AP and PA currents at all times following the WS. Voluntary rms EMG (colored bars, right *y*-axis) measured before the TMS pulse is shown normalized to values at the WS, and was similar for PA and AP currents across different time points for right hand-responding (***A***) and nonresponding (***B***) blocks. ***C***, MEP latencies measured at the WS were longer for AP currents compared with PA currents in both right hand-responding and nonresponding blocks. *p < 0.05, **p < 0.01, ***p < 0.001, compared with WS time point within each current direction; +*p* < 0.05, ++*p* < 0.01, +++*p* < 0.001, AP vs PA.

The suppression of MEPs during the preparatory period of the simple reaction time task depended on which hand was responding: AP-evoked MEPs were preferentially suppressed when preparing a response with the right hand (Fig. 4*A*); whereas, both PA and AP MEPs were similarly suppressed during the preparation of left-hand responses (i.e., right hand was nonresponding; Fig. 4*B*). This was supported by the rmANOVA showing a significant hand × current direction × time interaction (Table 2). Follow-up paired *t* tests for right-hand responses revealed that AP-evoked MEPs were suppressed at the time of the IS and 35%_RT_ compared with those at the WS, and although there appeared to be a small suppression of PA MEPs, the comparison did not survive the Bonferroni correction (Fig. 4*A*). At 70%_RT_ both PA and AP MEPs were facilitated (Fig. 4*A*). Additionally, comparison of normalized MEP amplitudes indicated a greater suppression of AP MEPs at the time of the IS and at 35%_RT_. This pattern of results is similar to those obtained for right-hand responses in the choice reaction time task (experiment 2; Fig. 3*A*). When the right hand was the nonresponding hand, paired *t* tests revealed that AP- and PA-evoked MEPs were suppressed at the WP (PA MEPs only), IS, 35%_RT_, and 70%_RT_ compared with those evoked at the time of the WS (Fig. 4*B*). There were no differences between PA and AP MEP amplitudes at any time point.

#### MEP latency

The latency of MEPs assessed at the time of the WS was greater for AP-evoked MEPs than PA-evoked MEPs for both right hand-responding and nonresponding trials (Fig. 4*C*). This was supported by a main effect of current direction in the rmANOVA (Table 2) and, again, highlighted the selective recruitment of PA and AP inputs. There was also a main effect of hand (Table 2), indicating that MEP latencies were slightly longer (0.2 ms on average) in right hand-responding versus nonresponding trials.

#### Voluntary rms EMG amplitude

The voluntary rms EMG amplitude in the right hand was generally consistent across current directions, right hand-responding and nonresponding trials, and time points (Fig. 4*A*,*B*), as indicated by a lack of main effects or interactions in the rmANOVA (Table 2).

#### Reaction time

Reaction times during the simple reaction time task were influenced both by the responding hand and the time of the TMS pulse (see Fig. 6*B*), as indicated by a significant hand × time interaction (Table 2). Follow-up paired *t* tests showed that, when collapsed across current directions, reaction times were shortened when TMS was delivered at the IS and 35%_RT_ compared with TMS delivered at the WS for right hand-responding and nonresponding trials (all *p* ≤ 0.01), and at 70%_RT_ for nonresponding trials (*p* < 0.01; see Fig. 6*B*).

### Experiment 4: Go/No Go task with directional TMS

#### MEP amplitude

**Figure 5.**
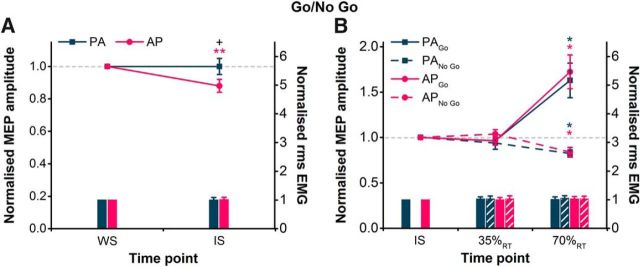
***A***, ***B***, During the Go/No Go task, MEP amplitudes in the right FDI, shown normalized to the WS time point (colored lines, left *y*-axis), were suppressed more for AP currents than for PA currents at the IS compared with the WS (***A***), indicating a selective anticipatory suppression in response to the WS. However, during successful No Go trials of the Go/No Go task, MEP amplitudes normalized to the IS were suppressed to a similar extent for AP currents as for PA currents at 70%_RT_ when compared with those at the IS (***B***), indicating a similar reactive suppression in response to the No Go signal. The facilitation of MEPs in Go trials at 70%_RT_ was similar for both current directions. Voluntary rms EMG measured before the TMS pulse (colored bars, right *y*-axis) is shown normalized to values at the WS (***A***) and IS (***B***), and was similar for PA and AP currents across different time points for Go and No Go trials. *p < 0.05, **p < 0.01, compared with IS time point within each current direction; +*p* < 0.05, AP vs PA.

We first assessed whether a selective preparatory suppression of AP MEPs was observed at the IS. There were no main effects of current direction or time; however, there was a significant current direction × time interaction (Table 3). *Post hoc* paired *t* tests revealed no difference in the absolute amplitude of PA and AP MEPs at WS (Table 1, *p* = 0.47). However, MEPs were suppressed at the IS compared with the WS for AP currents, but not PA currents (Fig. 5*A*). Furthermore, a paired *t* test on the normalized (to WS) amplitude of MEPs at the IS further illustrated greater suppression of AP-evoked compared with PA-evoked MEPs (Fig. 5*A*). The suppression of AP MEPs here is less than half of that observed in the CRTT (experiment 2) and SRTT (experiment 3), and could be a consequence of the longer warning period used here to minimize preparatory inhibition and emphasize reactive inhibition or could reflect the different task requirements.

**Table 3.** Results of rmANOVAs conducted for experiment 4

	Experiment 4: Go/No Go (*n* = 12)			
	WS vs IS (preparatory)		35%_RT_ and 70%_RT_ (after the IS)	
	*F*_[df,error]_	*p*	*F*_[df,error]_	*p*
MEP amplitude				
Trial type			29.750_[1,11]_	<0.001
Current direction	0.039_[1,11]_	0.847	1.147_[1,11]_	0.307
Time	2.805_[1,11]_	0.122	16.925_[1,11]_	0.002
Current direction × time	8.05_[1,11]_	0.016	0.157_[1,11]_	0.700
Trial type × time			27.276_[1,11]_	<0.001
Trial type × current direction			0.102_[1,11]_	0.755
Trial type × current direction × time			0.810_[1,11]_	0.387
Voluntary rms EMG amplitude				
Trial type			1.071_[1,11]_	0.323
Current direction	0.021_[1,11]_	0.888	0.483_[1,11]_	0.501
Time	0.057_[1,11]_	0.816	0.291_[1,11]_	0.600
Current direction × time	0.045_[1,11]_	0.836	0.049_[1,11]_	0.829
Trial type × time			0.035_[1,11]_	0.856
Trial type × current direction			0.088_[1,11]_	0.772
Trial type × current direction × time			1.577_[1,11]_	0.235
Reaction time				
Current direction			0.214_[1,11]_	0.653
Time			50.402_[3,33]_	<0.001
Current direction × time			2.344_[3,33]_	0.091

For the second analysis, we were interested in whether the suppression after the IS in the No Go condition was different between AP- and PA-evoked MEPs. The amplitude of MEPs at 35%_RT_ and 70%_RT_ was therefore normalized to those at the IS. Results showed that AP and PA MEPs were suppressed to a similar extent at 70%_RT_ in successful No Go trials and, as expected, were facilitated to a similar extent in the Go trials at 70%_RT_ (Fig. 5*B*). Three-way rmANOVA revealed the main effects of trial type and time and a significant trial type × time interaction (Table 3). There was no main effect of current direction or any interactions involving current direction (Table 3). *Post hoc* paired *t* tests on the pooled AP and PA MEPs indicated a significant suppression of MEPs at 70%_RT_ compared with the IS for No Go trials (*p* = 0.031), and a significant facilitation in Go trials (*p* = 0.011).

#### MEP latency

A paired *t* test on MEP latencies at the WS showed them to be significantly greater for AP (23.3 ± 0.5 ms) than PA MEPs (22.1 ± 0.5 ms; *p* < 0.001).

#### Voluntary rms EMG amplitude

The level of volitional muscle activity was analyzed in the same manner as that for MEP amplitudes, and it was found to be consistent across different current directions, trial types, and time points (Fig. 5*A*,*B*). First, two-way rmANOVA revealed no main effects of current direction or time, or an interaction of current direction × time (Table 3). Subsequent three-way rmANOVA revealed no main effects of current direction, trial type, or time, or any interactions (Table 3).

#### Reaction time

**Figure 6.**
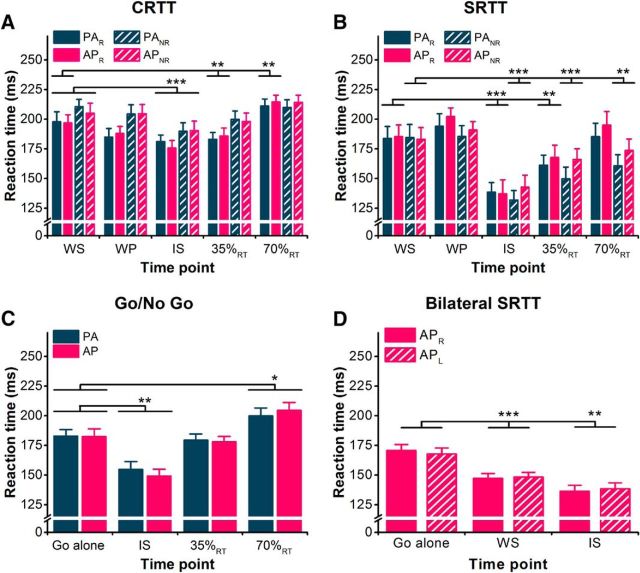
***A–D***, Mean EMG-determined reaction times shown for correct response trials and both PA and AP current directions in CRTT (***A***), SRTT (***B***), Go/No Go (***C***), and bilateral SRTT tasks (***D***). For the legends in ***A*** and ***B***, subscript R denotes right hand-responding trials and subscript NR denotes right-hand nonresponding trials (i.e., reaction times determined from the left hand). For the legend in ***D***, subscript R and L denote right-hand and left-hand responses in the same trial. *p < 0.05, **p < 0.01, ***p < 0.001, compared with WS time point in ***A*** and ***B*** and to Go alone (***C***, ***D***).

Reaction times were affected by the time at which TMS pulses were delivered (Fig. 6*C*). Two-way rmANOVA showed a main effect of time, but no effect of current direction or interaction of current direction × time (Table 3). Compared with the Go alone trials with no TMS, paired *t* tests showed that RTs were significantly shortened when TMS was delivered at the IS (*p* < 0.001) and increased when delivered at 70%_RT_ (*p* = 0.014).

### Experiment 5: relationship of reaction times and trial-by-trial variability in MEPs assessed with AP TMS

#### MEP amplitude

On average, MEPs in the right hand decreased by 28 ± 2% at the IS compared with the WS (*p* < 0.01).

#### Correlation between reaction times and MEP suppression

**Figure 7.**
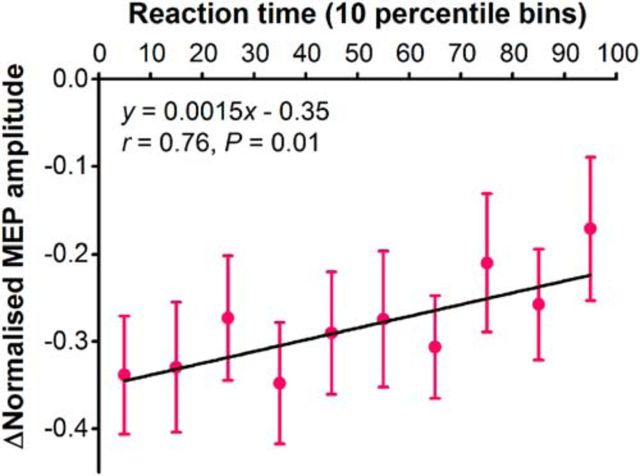
Correlation between mean MEP amplitude change and simple reaction time arranged in consecutive 10 percentile bins (e.g., 0 to 10th, 10th to 20th).

Greater preparatory suppression of AP-evoked MEPs at the IS was associated with slightly faster reaction times (Fig. 7). This was supported by a significant correlation at the group level between reaction time percentile bin and average MEP amplitude change (Fig. 7). Significant positive correlations were observed at the individual level in 6 of 11 participants, with no significant correlation being observed in the remaining 5 participants.

#### Reaction time

Reaction times were affected by the time at which TMS pulses were delivered (Fig. 6*D*). Two-way rmANOVA showed a main effect of time (*F*_(2,20)_ = 35.34, *p* < 0.001), but no effect of response hand (*F*_(1,10)_ = 0.00, *p* = 0.99), indicating that the reaction times were faster with TMS (WS and IS) compared with without (Go alone). There was a significant interaction of response hand × time (*F*_(2,20)_ = 4.64, *p* = 0.022), but *post hoc* tests revealed no differences between hands at any time (all *p* ≥ 0.14), and the mean difference at each time point was extremely small (±3 ms), so the meaningfulness of this is questionable.

## Discussion

### Selective inhibition of synaptic inputs to corticospinal neurons during movement preparation

These experiments made use of the fact that TMS can activate different sets of excitatory I-wave inputs to the corticospinal neurons. The novel finding is that, if the muscle is potentially involved in a forthcoming movement, late I-waves are selectively suppressed between the warning and imperative signal, while early I-waves are unaffected. Experiment 1 provided evidence for this using the H-reflex conditioning technique (van der Linden and Bruggeman, 1993; Niemann et al., 2017). At the time of the “Go” cue, H-reflex facilitation was reduced at long afferent–corticospinal volley delays, which we interpret as reflecting a reduced contribution of late I-waves to the overall facilitation of spinal motoneurons. We then corroborated this by comparing the responses to PA and AP TMS using our new method (D'Ostilio et al., 2016; Hannah and Rothwell, 2017) and showed that AP MEPs were selectively inhibited while PA MEPs were largely unchanged. These effects were observed in a right/left choice reaction time task (experiment 2), a simple reaction task in which the right hand always responded (experiment 3), and the Go/No Go task (experiment 4). The results suggest that when the timing of the imperative stimulus is highly predictable, selected inputs to the corticospinal neurons are suppressed rather than suppressing the whole of the output pathway. We conclude that the data rule out the simplest version of the subthreshold hypothesis that postulates that inhibition prevent premature release of excitatory inputs to corticospinal neurons. They are more compatible with more nuanced hypotheses of the role of inhibition in which there is a change in the balance of excitatory input to corticospinal neurons, rather than a simple inhibitory gating of corticospinal output. When the imperative signal occurs, the population activity evolves into a state where there is net facilitation of all inputs to corticospinal neurons, which results in a similar facilitation of PA and AP MEPs near to the onset of movement (70%_RT_).

At first sight, the results of our PA and AP TMS experiments might seem to contradict those of previous studies, which reported that PA-evoked MEPs were suppressed during the warning period of reaction time tasks (Hasbroucq et al., 1997; Touge et al., 1998; Duque and Ivry, 2009; Greenhouse et al., 2015). Our explanation for the previous results is that PA currents are not very selective in their recruitment of particular I-wave inputs, and thus PA MEPs, particularly when evoked using the high stimulus intensities needed at rest, must be generated by a mixture of both early and late I-wave activity. The effects seen in previous experiments were therefore likely due to a reduced contribution of late I-waves to the generation of PA MEPs. The results of our H-reflex conditioning experiment, performed at rest with subthreshold PA currents, are fully compatible with this explanation. In fact, there was a suggestion of weak suppression of PA MEPs when preparing for a right-hand response in experiment 3, which also supports this idea. The trick in our experiments is that brief AP currents are quite specific in their recruitment of late I-waves (Hannah and Rothwell, 2017), and so the comparison with PA-evoked MEPs allows us to dissociate changes in the relative excitability of early and late input pathways. Our interpretation relies on the assumption that the neural subpopulations recruited by PA and AP currents are equally sensitive to the tonic muscle contraction used to lower motor thresholds in the latter experiments. While we did not measure both RMTs and AMTs in the same experiment here, our unpublished observations based on a previous dataset (D'Ostilio et al., 2016) suggest that the PA 120 μs and AP 30 μs pulses show similar relative reductions in threshold from rest to muscle contraction (17% and 14%; *p* = 0.14). Thus, it seems unlikely that the present results could be explained by differential effects of muscle activity on PA- and AP-sensitive neuronal subpopulations.

A potential concern when evaluating changes in MEP size is that the site of any changes could be located at a cortical or a spinal level. There is evidence of concurrent changes in the spinal H-reflex as well as in MEPs during the warning period of reaction time tasks (Duque et al., 2010), implying that changes in spinal excitability could contribute to the smaller MEP. However, three features suggest that the selective inhibition of AP MEPs described here is of cortical origin. First, the main difference between current orientations is thought to be in how they activate corticospinal neurons in M1 (Day et al., 1989a; Hanajima et al., 1998; Di Lazzaro and Rothwell, 2014). Second, the latency differences between PA and AP currents can be observed in the same motor unit (Day et al., 1989b; Sakai et al., 1997; Hanajima et al., 1998; Hannah and Rothwell, 2017), so that any inhibition at the spinal level would be expected to affect AP and PA MEPs in the same way. Finally, and in line with recent data (Lebon et al., 2016), we found no evidence that the unconditioned H-reflex was suppressed in the warning period during an SRTT, which argues against a major role for spinal mechanisms in the suppression of the MEP under the present conditions.

### Broad inhibition of synaptic inputs to corticospinal neurons during outright response suppression

In contrast to the selective inhibition of AP MEPs, we also found evidence for the suppression of both PA and AP MEPs in the right FDI when a response of the right index had to be completely suppressed or aborted. These effects were observed soon after the warning stimulus in blocks of the SRTT where only a left index response was being prepared and the right index was response irrelevant (experiment 3, nonresponding). Note that this contrasts with the selective suppression of AP MEPs in the nonresponding hand during the CRTT. The similar suppression of PA and AP MEPs was also observed after the imperative signal (70%_RT_) in trials where the right index is response relevant but the No Go signal indicated that the initiation of a prepared response of the right index had to be stopped (experiment 4). This suggests that when the situation demands that a response must be suppressed, whether or not it is known in advance of the imperative, there is a broad suppression of corticospinal output that affects response-relevant and response-irrelevant muscle representations, as well as early and late I-wave inputs in both output zones.

It perhaps seems surprising that there was preparatory inhibition of the right FDI in a task that only involved a response of left index (experiment 2, nonresponding). The most likely explanation is that in the present experiments participants had to maintain a slight background contraction of both left and right FDI muscles (to lower the threshold for stimulation), and so the right FDI was still relevant for the task. Inhibition in this case might prevent potential mirror movements in the right index when preparing a response with the left index (Duque et al., 2005). Alternatively, Greenhouse et al. (2015) recently suggested that broad suppression of the motor system was a general feature of the response preparation process that helped resolve “competition resolution” by reducing noise to enhance signal processing and in turn enhance the gain of a selected response. This argument cannot fully explain our results, however, since we saw a differential regulation of PA and AP MEPs, depending on whether the right index was response relevant or irrelevant (experiment 3, responding vs nonresponding).

The contrast between targeted inhibition of specific inputs to corticospinal neurons and broader inhibition of both input pathways was illustrated particularly well in the Go/No Go task (experiment 4). Selective inhibition of AP MEPs at the time of the imperative signal was replaced by the inhibition of both PA and AP MEPs after the IS during successful response cancellation in No Go trials. The less selective inhibition when completely suppressing a response might be suggestive of somatic inhibition of the corticospinal neurons.

### Functional significance of motor cortex inhibition

The results of experiment 5 demonstrated a relationship between the extent of preparatory inhibition of MEPs and response times. We found that greater preparatory suppression of the corticospinal pathway was associated with slightly faster reaction times. Importantly, experiment 5 was similar to experiment 3 in that it involved response preparation with the index fingers of both the left and right hands. In both cases, inhibition seems to target a specific set of inputs to the corticospinal neurons (late I-waves), rather than the corticospinal neuron cell body. These data seem to argue against the hypothesis that preparatory inhibition of M1 output neurons serves to brake the initiation of the movement being prepared (Touge et al., 1998; Duque and Ivry, 2009), since one might have expected preparatory inhibition to slow response times. However, they would be highly compatible with the dynamical systems concept that the coexistence of balanced excitation and inhibition is an essential part of successful movement preparation. They also fit well with recent data showing that in addition to neurons showing excitation, there is a specific population of layer II–III neurons in mouse motor cortex that is suppressed during the waiting period before movement (Hasegawa et al., 2017). In fact, the amount of suppression correlated well with reaction time.

Cancelling a movement altogether, as in the nonresponding/No-Go trials of experiments 3 and 4, seems to involve a process that is different from the coordinated change in activity patterns described above and, instead, might rely on the direct suppression of M1 corticospinal output neurons. This would be akin to an inhibitory gate that prevents any buildup of excitatory activity from driving corticospinal neurons and thus causing unwanted movement.

### Conclusions

The experiments suggest that premovement suppression of MEPs is not caused by the suppression of corticospinal output that prevents premature release of an excitatory motor command. Instead, it seems to affect only specific inputs to the corticospinal system and is compatible with the idea that the suppression of specific sets of cortical neurons is an essential part of successful movement preparation.
